# Associations among ancestry, geography and breast cancer incidence, mortality, and survival in Trinidad and Tobago

**DOI:** 10.1002/cam4.503

**Published:** 2015-09-04

**Authors:** Wayne A Warner, Robert L Morrison, Tammy Y Lee, Tanisha M Williams, Shelina Ramnarine, Veronica Roach, Simeon Slovacek, Ravi Maharaj, Nigel Bascombe, Melissa L Bondy, Matthew J Ellis, Adetunji T Toriola, Allana Roach, Adana A M Llanos

**Affiliations:** 1 Oncology Division, Washington University School of MedicineSt. Louis, Missouri; 2 Siteman Cancer Center, Washington University School of MedicineSt. Louis, Missouri; 3 California State UniversityLos Angeles, California; 4 University of ConnecticutStorrs, California; 5 Department of Genetics, Washington University School of MedicineSt. Louis, Missouri; 6 Dr. Elizabeth Quamina Cancer Registry, Eric Williams Medical Sciences ComplexMt. Hope, Trinidad and Tobago; 7 Department of Clinical Surgical Sciences, Faculty of Medical Sciences, University of the West IndiesTrinidad and Tobago; 8 Lester and Sue Smith Breast Center, Baylor College of MedicineHouston, Texas; 9 Department of Surgery, Washington University School of MedicineSt. Louis, Missouri; 10 Public Health and Primary Care Unit, Department of Paraclinical Sciences, Faculty of Medical Sciences, University of the West IndiesTrinidad and Tobago; 11 Rutgers School of Public Health and Rutgers Cancer Institute of New Jersey, Rutgers UniversityNew Brunswick, New Jersey

**Keywords:** Ancestry, breast cancer, Caribbean, geography, incidence, mortality, survival, Trinidad and Tobago

## Abstract

Breast cancer (BC) is the most common newly diagnosed cancer among women in Trinidad and Tobago (TT) and BC mortality rates are among the highest in the world. Globally, racial/ethnic trends in BC incidence, mortality and survival have been reported. However, such investigations have not been conducted in TT, which has been noted for its rich diversity. In this study, we investigated associations among ancestry, geography and BC incidence, mortality and survival in TT. Data on 3767 incident BC cases, reported to the National Cancer Registry of TT, from 1995 to 2007, were analyzed in this study. Women of African ancestry had significantly higher BC incidence and mortality rates (Incidence: 66.96; Mortality: 30.82 per 100,000) compared to women of East Indian (Incidence: 41.04, Mortality: 14.19 per 100,000) or mixed ancestry (Incidence: 36.72, Mortality: 13.80 per 100,000). Geographically, women residing in the North West Regional Health Authority (RHA) catchment area followed by the North Central RHA exhibited the highest incidence and mortality rates. Notable ancestral differences in survival were also observed. Women of East Indian and mixed ancestry experienced significantly longer survival than those of African ancestry. Differences in survival by geography were not observed. In TT, ancestry and geographical residence seem to be strong predictors of BC incidence and mortality rates. Additionally, disparities in survival by ancestry were found. These data should be considered in the design and implementation of strategies to reduce BC incidence and mortality rates in TT.

## Introduction

As the most commonly diagnosed malignancy among women worldwide, breast cancer (BC) is clearly a major global health concern. The most recent data from the International Agency for Research on Cancer (IARC) estimated that 1.67 million incident BC cases were diagnosed in 2012 1. While age-standardized incidence rates vary extensively, approximately 53% of all incident BC cases occurred in economically developing countries. Globally, BC is the fifth most common cause of cancer mortality; whereas, in developed countries, it is the second leading cause of mortality among women, in economically developing countries this malignancy is the most prevalent cause of cancer mortality 1.

The vast majority of studies to date have focused on BC in developed countries, where incidence and mortality rates have demonstrated a decreasing trend (or at least remaining stable in many countries). In contrast, studies are limited among many developing countries, where incidence and mortality are projected to continually increase in the next few decades 2–5. This is particularly pronounced in developing countries of the Caribbean where population growth, aging, increasing life expectancy 5, and the adoption of “westernized” lifestyles, are identified as possible contributing factors to the increasing incidence 3,6,7. Furthermore, lack of access to adequate comprehensive breast care, including mammography and timely, appropriate treatment and follow-up care, may contribute to increases in BC mortality 8,9.

One such country is the English-speaking Republic of Trinidad and Tobago (TT) situated in the Southern Caribbean (just northeast of Venezuela). Designated by the World Bank 10 as a high-income economy, given that it has a gross national income (GNI) of ≥$12,746, TT has not reached the final stage of development. Nonetheless, TT is the most industrialized of all Caribbean nations, with oil, natural gas, chemical industries, and food and beverage industries, and has recently experienced an 8% economic growth, more than double the regional average 11. As of 2011 12, TT had a population of 1,328,019, with diverse self-reported ancestries, including East Indian (35%), African (34%), mixed (23%; [African/East Indian, 8% and mixed/other, 15%]), other (<1%), and unknown ancestries (6%) 12.

The Ministry of Health is the national authority responsible for oversight of the national healthcare system. There are five Regional Health Authorities (RHAs) that are responsible for the provision of healthcare services in their respective geographic catchment area. Cancer screening and treatment services are provided free to all citizens of TT at six public medical facilities. Furthermore, the Chronic Disease Assistance Programme provides free oncology prescription drugs.

Despite the opportunities afforded by universal healthcare in TT, BC mortality rates are among the highest in the Caribbean and the world 13–16. A recent study by Camacho-Rivera and colleagues 16, indicated that women in TT are diagnosed with BC at a later stage and initiate/receive multi-mode therapy (i.e., combination of ≥2 types of treatment) for their disease at very low levels 16. Utilizing data from the National Cancer Registry of TT, on incident BC cases diagnosed between January 1995 and December 2007, we sought to expand upon the findings of Camacho-Rivera and colleagues 16, with the objective of investigating associations among ancestry, geography and BC incidence, mortality and survival in TT. Findings from this study will be integral to understanding some of the factors associated with the poor outcomes observed among BC patients in TT.

## Materials and Methods

### Study design and measures

This study included incident, invasive BC cases among female patients reported to the National Cancer Registry of TT. Prospectively collected tumor incidence and mortality data from January 1, 1995 and December 31, 2007 were included in the analysis. The population-based cancer registry was established in 1994 by the Trinidad and Tobago Cancer Society, using cancer registry guidelines set by IARC 17,18. This registry uses the CANREG database and statistical software (Version 4.33). Records from both public and private biomedical institutions populated the registry dataset. Public sector institutions included Port of Spain General Hospital, Caura Hospital, National Radiotherapy Center, Sangre Grande Hospital, Tobago Regional Hospital, Mount Hope Women’s Hospital, Eric Williams Medical Sciences Complex, San Fernando Hospital, Point Fortin Area Hospital, and the Central Statistical Office. Private sector data sources included Augustus Long Hospital, Petrotrin-Santa Flora Medical Centre, Community Hospital of the Seven Day Adventists, Brian Lara Treatment Centre, and Westshore Private Hospital.

**Figure 1:**
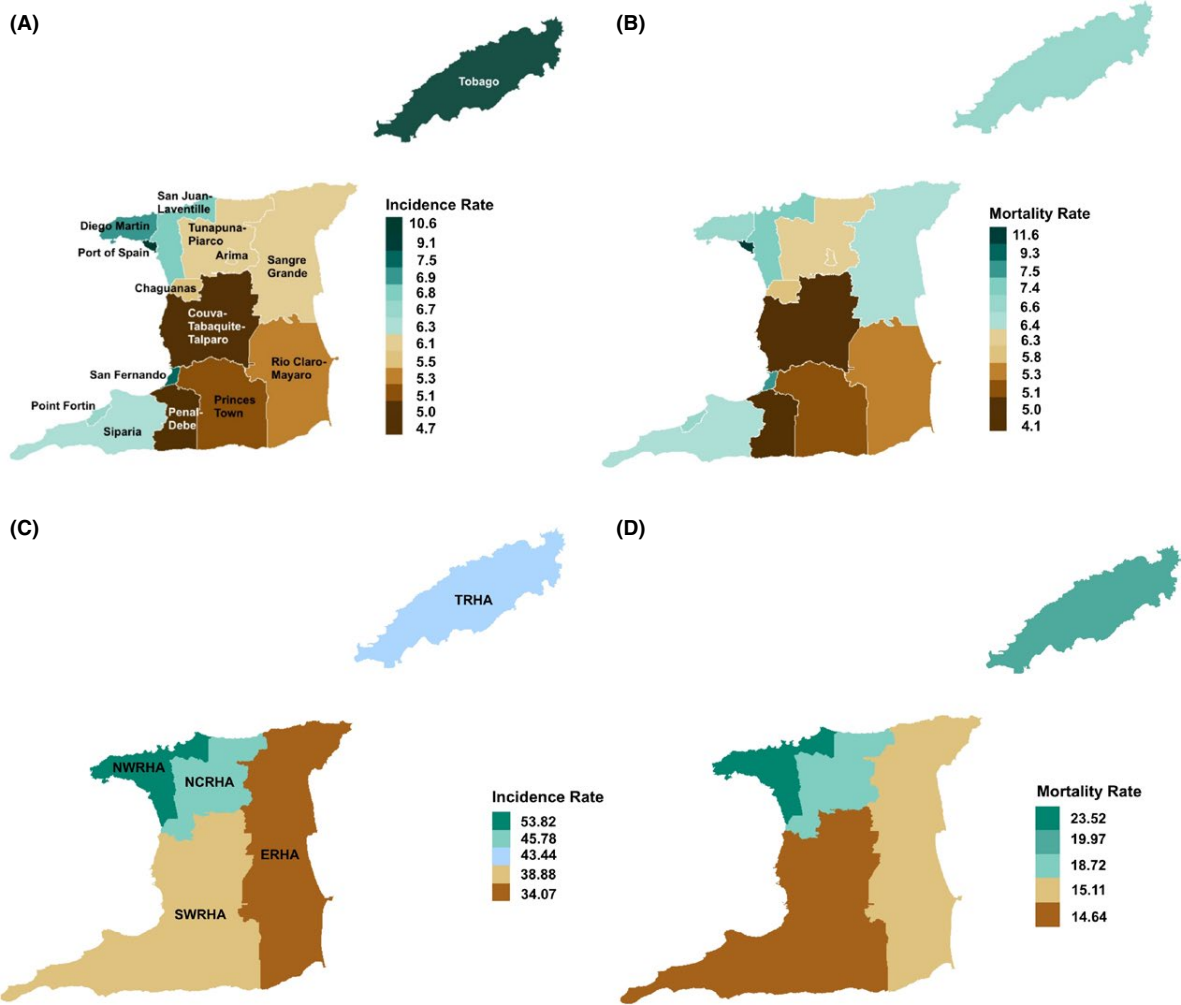
Geospatial maps of breast cancer incidents and mortality rates in Trinidad and Tobago 1995–2007: (A–B) age-standardized rates for all corporations, and (C–D) age-standardized rates for all Regional Health Authorities.

In Trinidad, there is a local government system with five Municipal Corporations for the cities of Port of Spain and San Fernando, and the boroughs of Arima, Point Fortin and Chaguanas, as well as nine Regional Corporations (including those of: (1) Couva, Tabaquite, and Talparo; (2) Diego Martin; (3) Mayaro and Rio Claro; (4) Penal and Debe; (5) Princes Town; (6) Sangre Grande; (7) San Juan and Laventille; (8) Siparia; and (9) Tunapuna and Piarco) (Fig.1). Tobago is administratively controlled by the Tobago House of Assembly. The geographical area of residence data from the cancer registry was aligned with the corresponding Ministry of Health RHAs, which provides healthcare personnel and facilities for persons residing in the catchment area. The North West Regional Health Authority (NWRHA); North Central Regional Health Authority (NCRHA); South West Regional Health Authority (SWRHA); Eastern Regional Health Authority (ERHA) and Tobago Regional Health Authority (TRHA) are responsible for direct provision of healthcare services in their respective catchment area. However, there are specialized cancer centers in various RHAs that can be accessed with a doctor’s referral without regard to the patient’s place of residence.

In the registry data file, demographic characteristics (age at diagnosis and ancestry) were self-reported. Four ancestry groups are most prevalent among the population of TT: African, East Indian, mixed, and other. Imputation was used to ascribe ancestry among cancer cases with unknown/missing ancestry (*n* = 5301 [21.2%]) 19. Essentially, four separate predictive binary logistic regression models were calculated. Ancestry was used as the dependent variable for estimation purposes. Known demographic variables including: gender, age, and residence (15 corporations and boroughs used as dummy variables) were used as independent variables in the four predictive regression models to assign ancestry based on known profiles from all instances of self-reporting data. After fitting the four models for the cases with missing ancestry data, probabilities of predicted values of ancestry were calculated. These values revealed the probability of each cancer patient’s placement into each of the four ancestry groups. Cancer patients with missing ancestry were then assigned to one of the four ancestry groups according to the highest probability of being a member of that group. Using this method, all of the cases with unknown/missing ancestry were assigned to either the African or East Indian ancestry group as the other probabilities were significantly lower and would have increased the errors of estimation.

Clinical data (stage, grade, histology, and treatment modalities) were classified based on WHO International Classification of Disease (ICD-O-3) codes. Female BC cases with missing data and/or data errors (e.g., date of death before date of incident BC, no date of last follow-up, etc.) were excluded (*n* = 10). The analytic dataset consisted of 3767 incident invasive BC cases. This study received approval by Institutional Review Boards of all participating institutions.

### Statistical analysis

Study sample characteristics overall and indexed by ancestry were described using frequencies and proportions. Chi-square and Fisher’s exact tests were used to compare sample characteristics by ancestry. Crude incidence and mortality rates in TT were estimated using population data from the TT 2000 and 2010 census 12. From these estimates, we calculated the age-standardized incidence and mortality rates (per 100,000) based on the world-standardized population weights 20, to better compare BC rates in TT to global trends. Populations of corporations in the other years were estimated through interpolation using the “irregular points of year” estimation method 17,18. Cox proportional hazards models were used to calculate the hazard ratios (HR) and 95% confidence intervals (CIs) of BC mortality by ancestry and by geography. In the initial model, we adjusted for age at BC diagnosis only. In the multivariable-adjusted model, we further adjusted for marital status, mode of detection, cancer stage, and whether any BC treatment was received. All reported *P*-values are two-sided and *P *< 0.05 was considered statistically significant. Analyses were done using Statistical Package of Social Science V.20 (SPSS) (IBM Corporation, Valhalla, NY) and R Statistical Software (R Foundation, Vienna, Austria).

## Results

### Characteristics of incident BC cases in Trinidad and Tobago

Data are reported on 3767 incident primary BC cases (54% of African ancestry, 33% of Indian ancestry, and 13% of mixed ancestry) reported to the TT cancer registry between January 1, 1995 and December 31, 2007. As shown in Table1, overall the mean age at diagnosis was 56 years, with significant differences by ancestry. Specifically, women of Indian ancestry presented at a younger age, compared to women of African ancestry or mixed ancestry (*P *< 0.01).

**Table 1:** Characteristics of incident breast cancer cases reported to the cancer registry, overall and by ethnicity, Trinidad and Tobago, 1995–2007

Characteristics	Overall *N* = 3767 *n* (%)	African *n* = 2020 *n* (%)	Indian *n* = 1246 *n* (%)	Mixed *n* = 501 *n* (%)	*P* 1
*Demographic characteristics*					
Age at diagnosis (years), mean ± SD	56.55 ± 14.63	57.46 ± 15.29	54.29 ± 13.00	58.48 ± 15.10	**<0.01**
Age at diagnosis (years)					
<45	856 (22.72)	451 (22.33)	307 (24.64)	98 (19.56)	**<0.01**
45–60	1489 (39.53)	743 (36.78)	544 (43.66)	202 (40.32)	
>60	1422 (37.75)	826 (40.89)	395 (31.70)	201 (40.12)	
Marital status					
Divorced/separated/widowed	727 (19.30)	375 (18.56)	240 (19.26)	112 (22.36)	**<0.01**
Married/living as married	1669 (44.31)	751 (37.18)	693 (55.62)	225 (44.91)	
Single/never married	744 (19.75)	499 (24.70)	133 (10.67)	112 (22.36)	
Unspecified	627 (16.64)	395 (19.55)	180 (14.45)	52 (10.38)	
Geographic area of residence^2^					
Eastern	194 (5.15)	100 (4.95)	68 (5.46)	26 (5.19)	**<0.01**
North Central	915 (24.29)	457 (22.62)	333 (26.73)	125 (24.95)	
North West	1314 (34.88)	866 (42.87)	195 (15.65)	253 (50.50)	
South West	1175 (31.19)	442 (21.88)	643 (51.61)	90 (17.96)	
Tobago	165 (4.38)	152 (7.52)	6 (0.48)	7 (1.40)	
Unknown	4 (0.11)	3 (0.15)	1 (0.08)	0 (0.00)	
*Clinical characteristics*					
Mode of detection					
Clinical diagnosis	1601 (42.5)	849 (42.30)	534 (42.35)	218 (41.52)	**<0.01**
Incidental finding at autopsy	2 (0.05)	2 (0.10)	0 (0.00)	0 (0.00)	
Unknown/missing	2164 (57.45)	1156 (57.60)	727 (57.65)	281 (56.31)	
Stage at diagnosis					
Localized	1504 (39.93)	768 (38.02)	530 (42.54)	206 (41.12)	**<0.01**
Regional	1268 (33.66)	652 (32.28)	430 (34.51)	186 (37.13)	
Distant	323 (8.57)	202 (10.00)	79 (6.34)	42 (8.38)	
Unknown	672 (17.84)	398 (19.70)	207 (16.61)	67 (13.37)	
Grade					
Grade I – Well differentiated	68 (1.81)	37 (1.83)	30 (2.41)	1 (0.20)	**<0.01**
Grade II – Moderately Differentiated	175 (4.65)	97 (4.80)	56 (4.49)	22 (4.39)	
Grade III – Poorly Differentiated	467 (12.40)	256 (12.67)	127 (10.19)	84 (16.77)	
Grade IV – Undifferentiated	116 (3.08)	49 (2.43)	57 (4.57)	10 (2.00)	
Unspecified	2941 (78.07)	1581 (78.27)	976 (78.33)	384 (76.65)	
Histology					
Adenocarcinoma	350 (9.29)	164 (8.12)	158 (12.68)	28 (5.59)	**<0.01**
Carcinoma NOS	1068 (28.35)	629 (31.14)	306 (24.56)	133 (26.55)	
Other	2349 (62.36)	1227 (60.74)	782 (62.76)	340 (67.86)	
Surgical treatment received					
Yes	2778 (73.75)	1404 (69.50)	984 (78.97)	390 (77.84)	**<0.01**
No	502 (13.33)	311 (15.40	125 (10.03)	66 (13.17)	
Missing	487 (12.92)	305 (15.10)	137 (11.00)	45 (8.99)	
Chemotherapy initiated					
Yes	1576 (41.84)	779 (38.56)	567 (45.50)	230 (45.91)	**<0.01**
No	1699 (45.10)	933 (46.19)	542 (43.50)	224 (44.71)	
Missing	492 (13.16)	308 (15.25)	137 (11.00)	47 (9.38)	
Radiation therapy initiated					
Yes	1355 (35.97)	692 (34.26)	433 (34.75)	230 (45.91)	**<0.01**
No	1259 (33.42)	688 (34.06)	423 (33.95)	148 (29.54)	
Missing	1153 (30.61)	640 (31.68)	390 (31.30)	123 (24.55)	
Hormone therapy initiated					
Yes	1282 (34.03)	647 (32.03)	451 (36.20)	184 (36.73)	**<0.01**
No	1995 (52.96)	1067 (52.82)	658 (52.81)	270 (53.89)	
Missing	490 (13.01)	306 (15.15)	137 (11.00)	47 (9.38)	
Immunotherapy initiated					
No	3270 (86.81)	1709 (84.60)	1109 (89.00)	452 (90.22)	**<0.01**
Missing	494 (13.11)	310 (15.35)	137 (11.00)	47 (9.38)	
Vital status at last contact					
Alive	2306 (61.22)	1141 (56.49)	842 (67.58)	323 (64.47)	**<0.01**
Deceased	1461 (38.78)	879 (43.51)	404 (32.42)	178 (35.53)	

NOS, not otherwise specified. Statistically significant (P <0.05) differences are bolded.

1 Chi-square or Fisher’s exact tests were used to derive *P*-values.

2 Geographic area of residence was based on residence within the catchment area of the five Regional Health Authorities (RHAs), which are responsible for the provision of healthcare services in Trinidad and Tobago.

In terms of clinical characteristics, localized stage (39.93%) was most frequent, followed by regional (33.66%) and distant (8.57%) stages, with the highest frequency of BC patients diagnosed with localized cancer observed among women of Indian ancestry (*P *< 0.01). Although there were substantial ‘unspecified’ data on tumor grade (78% overall), women of mixed ancestry were more frequently diagnosed with poorly differentiated tumors and least likely to be diagnosed with well-differentiated tumors, compared to women of African or Indian ancestry (*P *< 0.01). Histologically, adenocarcinoma or carcinoma, not otherwise specified were diagnosed less frequently among women of mixed ancestry (32.14%), compared to women of Indian (37.24%) or African ancestry (39.26%; *P *< 0.01). There were significant differences in the receipt of surgical treatment and initiation of chemotherapy, radiation therapy, hormone therapy, and immunotherapy by ancestry; specifically, women of African ancestry were least likely to have received/initiated any BC treatment compared to women of Indian and mixed ancestry (all *P* < 0.01). At the time of last contact (1997–2007), the largest proportion of cases still alive were of Indian ancestry (67.58%), followed by those of mixed ancestry (64.47%), and lastly, African ancestry (56.49%).

### Breast cancer incidence, mortality, and survival in Trinidad and Tobago

Overall, women of African ancestry had the highest age-standardized BC incidence rates per 100,000, followed by women of Indian and mixed ancestry (African ancestry: 66.96, 95% CI 63.95–65.97; Indian ancestry: 41.04, 95% CI 38.75–43.34; and mixed ancestry: 36.72, 95% CI 33.39–40.03). Women of African ancestry also had the highest age-standardized BC mortality rates (30.82 per 100,000, 95% CI 28.80–32.84), followed by women of Indian ancestry (14.19 per 100,000, 95% CI 12.84–15.54) and mixed ancestry (13.80 per 100,000, 95% CI 11.79–15.81).

As shown in Figure1, BC incidence and mortality rates were highest in the NWRHA geographic region. In NWRHA, incidence and mortality rates were 53.82 and 23.52 per 100,000, respectively; the nation’s capital city of Port-of-Spain had the highest rates of both incidence (75.19 per 100,000) and mortality (36.12 per 100,000). In NCRHA, incidence and mortality rates were 45.78 per 100,000 and 18.72 per 100,000, respectively; the corporation of Arima had the incidence (66.13 per 100,000) and mortality rates (27.34 per 100,000). In TRHA, incidence and mortality rates were 43.44 and 19.97 per 100,000; the parish of St. David had the highest incidence (58.60 per 100,000) and mortality rates (28.00 per 100,000), respectively. In SWRHA, incidence and mortality rates were 38.88 and 14.64 per 100,000. In the SWRHA area, the corporation of San Fernando had the highest BC incidence (47.12 per 100,000) and Point Fortin demonstrated the highest mortality rate (21.09 per 100,000). In ERHA, incidence and mortality rates were 34.07 and 15.11 per 100,000; the corporation of Sangre Grande had the highest incidence (35.28 per 100,000) and mortality rates (16.41 per 100,000).

Table2 shows the HRs and CIs of BC mortality by geography. After adjustment for age at incidence, marital status, detection method, cancer stage, and treatment initiation, the mortality rates in the NCRHA (HR 0.86, 95% CI: 0.75–0.98) and SWRHA (HR 0.67, 95% CI: 0.58–0.77) were significantly lower than observed in the NWRHA. We further investigated differences in mortality rates in each RHA by ancestry (Table3). Looking within the individual RHAs, compared to women of Indian ancestry, those of African ancestry had higher mortality rates in NCRHA (HR 1.27, 95% CI, 1.00–1.62) and NWRHA (HR 1.46, 95% CI, 1.14–1.87).

**Table 2:** Hazard ratios (HR) and 95% confidence intervals (CI) of mortality in each Regional Health Authority (RHA) catchment area in Trinidad and Tobago, 1995–2007

Mortality	NWRHA	ERHA	NCRHA	SWRHA	TRHA
Model					
Age-adjusted	1.00 (Ref)	1.06 (0.83–1.34)	0.94 (0.82–1.07)	**0.80 (0.70–0.91)**	0.98 (0.76–1.26)
Multivariable-adjusted^1^	1.00 (Ref)	0.98 (0.77–1.24)	**0.86 (0.75–0.98)**	**0.67 (0.58–0.77)**	0.98 (0.76–1.27)

Statistically significant (*P* <0.05) estimates are bolded.

1 Multivariable models adjusted for age at incidence, marital status, detection method, cancer stage, and treatment (yes/no). The model was adjusted for initiation of any treatment.

**Table 3:** Hazard ratios (HR) and 95% confidence intervals (CI) of mortality (B) in each Regional Health Authority (RHA) catchment area in Trinidad and Tobago, by ancestry, 1995–2007

Mortality	Indian	Mixed	African
All RHAs			
Age-adjusted	1.00 (Ref)	0.93 (0.78–1.11)	**1.31 (1.17–1.48)**
Multivariable-adjusted^1^	1.00 (Ref)	0.99 (0.83–1.19)	1.12 (0.99–1.26)
ERHA			
Age-adjusted	1.00 (Ref)	0.48 (0.19–1.17)	0.90 (0.55–1.49)
Multivariable-adjusted[Table-fn tf3-2]1	1.00 (Ref)	0.89 (0.35–2.25)	1.01 (0.59–1.74)
NCRHA			
Age-adjusted	1.00 (Ref)	1.26 (0.90–1.77)	**1.27 (1.00-1.62)**
Multivariable-adjusted^1^	1.00 (Ref)	1.12 (0.79–1.58)	0.97 (0.75–1.25)
NWRHA			
Age-adjusted	1.00 (Ref)	0.88 (0.65–1.20)	**1.46 (1.14–1.87)**
Multivariable-adjusted^1^	1.00 (Ref)	0.85 (0.63–1.16)	1.20 (0.94–1.55)
SWRHA			
Age-adjusted	1.00 (Ref)	0.74 (0.48–1.15)	1.16 (0.94–1.44)
Multivariable-adjusted^1^	1.00 (Ref)	0.98 (0.63–1.53)	0.91 (0.72–1.15)
TRHA			
Age-adjusted	1.00 (Ref)	0.57 (0.09–3.49)	0.67 (0.16–2.79)
Multivariable-adjusted[Table-fn tf3-2]	1.00 (Ref)	0.36 (0.05–2.53)	0.49 (0.12–2.12)

Statistically significant (*P* < 0.05) estimates are bolded.

1 Multivariable models adjusted for age at incidence, marital status, detection method, cancer stage, and treatment (yes/no).

**Figure 2:**
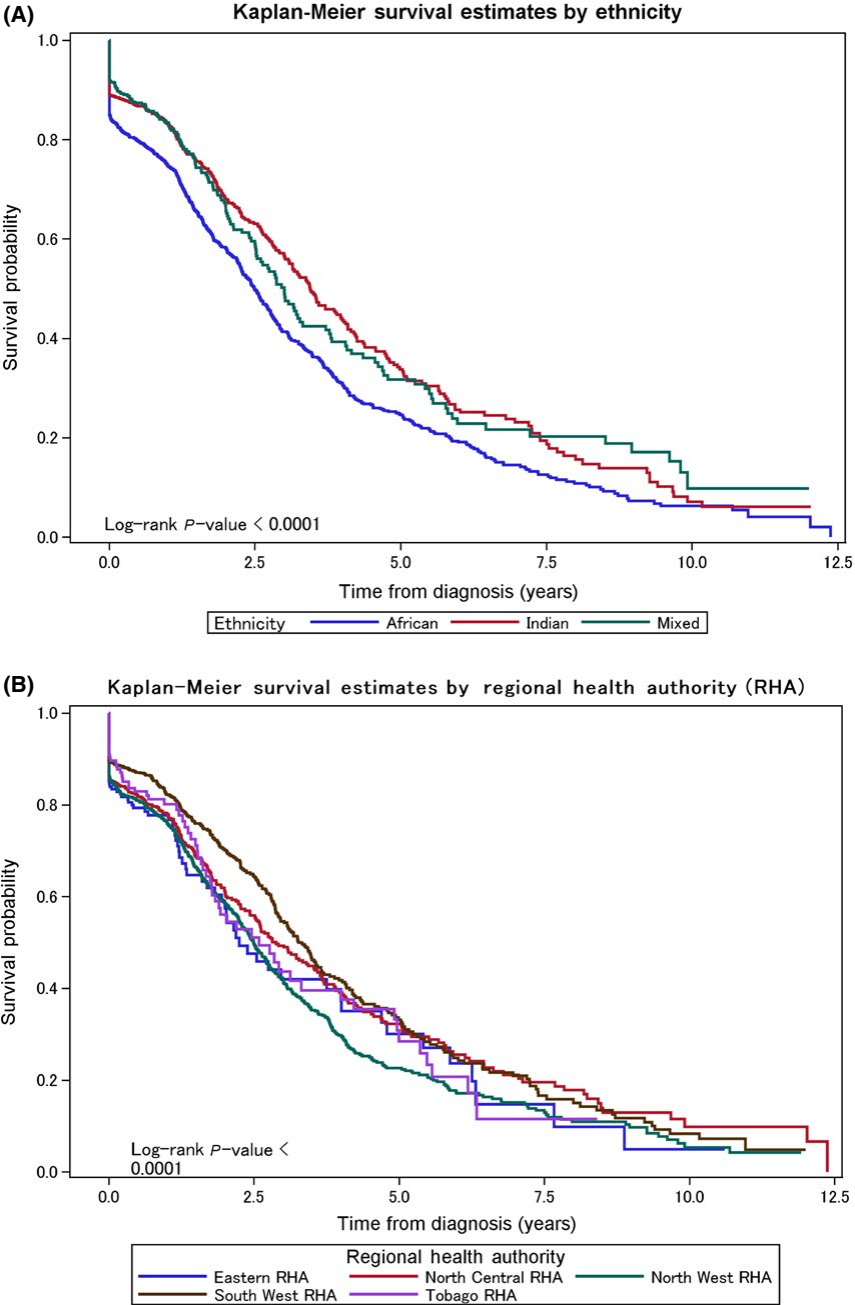
Breast cancer survival probability by ancestry (A) and by geography (B) based on residence within Regional Health Authority catchment area.

Unadjusted Kaplan–Meier curves indicate that BC cases residing in the NWRHA catchment area had lowest survival probability (5-year survival, 24%; 10-year survival, 5%). For women in the other RHAs, the 5-year survival rate was 30% and the 10-year survival rate was <20% (Fig.2A). Unadjusted Kaplan–Meier curves showed that the African ancestry was associated with the lowest survival probability over 12.5-year follow-up period, whereas women of Indian ancestry had the best survival probability up to approximately year 7.5 with mixed ancestry having the best survival probability beyond that year. The 5-year survival rates for patients of African, mixed and Indian ancestry were 24%, 32%, and 38%, respectively, and the 10-year rates were 5%, 5.1%, and 8%, respectively (Fig.2B).

## Discussion

Analysis of ancestral and geographic differences in BC incidence and mortality rates is an essential component of understanding the epidemiologic landscape of BC in TT. In this study, we investigated whether disparities existed by ancestry and geography for BC incidence, mortality, and survival in TT for the study period 1995–2007. We observed striking differences in these measures by ancestry and geography. Most notably, women of African ancestry had a BC incidence rate that was twice as high as that observed among women of East Indian and mixed ancestry. This finding was true in all geographic regions of TT. Overall, incidence and mortality rates were highest among residents of the NWRHA catchment area, which includes the capital city of TT, Port of Spain. Relatedly, BC cases residing in this geographic area also exhibited the lowest 5-year relative survival.

It is plausible that the highest BC incidence rates were observed in the NWRHA geographic area given that the majority of the public as well as private institutions offering cancer screening, diagnosis and/or treatment services are located in this catchment area including, the TT Cancer Society, Port of Spain General Hospital, National Radiotherapy Centre, the Brian Lara Cancer Treatment Centre of TT, and Pink Hibiscus Breast Health Specialists. What may account for the high mortality rates that were also observed in this area relates to the fact that between 1995 and 2007, the National Radiotherapy Center was the only public center offering adjuvant and palliative care, which we hypothesize would have reduced access to timely treatment among some BC patients. This merits further study to understand such factors as preventive care, outreach efforts, medical interventions, in particular genomic (personalized) medical treatments, and resource allocation throughout the nation.

In the United States (U.S.), minority women, particularly women of African descent, although having lower overall BC incidence, are more likely than their white counterparts to die from their disease, even after accounting for later stage at diagnosis 21–23. In contrast, what we have observed among BC cases in TT is that both incidence and mortality rates were highest among women of African descent. Similar findings were observed by Dindyal et al. 24, who analyzed data from the Port of Spain region of TT specifically and found that the largest proportion of BC was diagnosed among women of African descent (54%) followed by women of Indian descent (35%) and mixed ancestry (11%). Some studies have suggested that differences in BC incidence and mortality are related to factors related to socioeconomic status and/or inequity in healthcare access and utilization 25–29. While this may be true in the U.S., access to care is likely not a major concern in TT, given the equal access to healthcare model in place in the nation. However, it is quite possible that intrinsic biological variation in BC exists by ancestry or differences in the distribution of etiological factors by ancestry may explain some of the observed disparities in TT 30,31. In addition, BC among women of African ancestry tends to have more aggressive features 2,21,32. It is unknown whether this association exists among women in TT, therefore warranting further study.

The TT cancer registry database captured information on systemic therapies delivered to BC patients, however there was no clinical annotation containing hormone receptor expression status, BC subtype information, or indication as to whether BC treatment was administered in the adjuvant setting. A recent study examining BC treatment and outcomes in TT reported that >50% of BCs diagnosed between 1995 and 2005 were approximated to hormone receptor negativity, using receipt of hormone therapy as a surrogate 16. Intrinsic subtyping of BC patients at the time of diagnosis is not routinely done in TT. It may be that a high prevalence of BCs with more aggressive features (e.g., hormone receptor negativity, later stage, higher grade) may contribute to the high mortality and poor survival rates observed in TT 33,34. Given that BC is a heterogeneous disease, it is critical that molecular subtyping be prioritized in TT as this will allow for improved patient prognostication and stratification, and personalized treatment 22,33,35,36.

In many higher income countries, there has been a documented decline or leveling off of BC incidence rates 37. In the data from TT, however, we observed an increase across all demographic groups. These findings imply a need to improve cancer prevention, screening, and treatment options, as well as genetics and genomics-based research. In higher income countries, there is an increasing shift toward precision medicine, where the mutational landscape is evaluated to determine the biology of the tumor, risk of recurrence, and the most appropriate treatment protocol 37. TT will need to develop a core genetics and genomics-sequencing infrastructure to address the local genomic complexity of cancer.

Our finding of older age at diagnosis among women of African ancestry suggests that there might be underlying molecular mechanisms playing a central role. This is consistent with data from Raju et al. 38, which indicated that carcinoma was observed in only 1.1% of all breast biopsies among women ≤30 years. Still, almost one-quarter of incident BCs (22.72%) in this study were diagnosed at <45 years. It is well established that germline mutations in BC susceptibility genes such as *BRCA1*, *BRCA2*, *CHEK2*, and *TP53* have founder effects leading to increased risk for cancer incidence and mortality 39–45, particularly among younger women. Interestingly, there has also been a documented increase in BC mortality over the past few decades in TT. Naraynsingh and colleagues^46^ showed that between 1970 and 2004, age-standardized BC mortality had increased from 14.9 per 100,000 to 24.4 per 100,000, with an increase observed among women <50 years as well as those ≥50 years. Among women <50 years, mortality rates increased from 3.8 per 100,000 in 1970 to 9.2 per 100,000 in 2004, whereas among women ≥50 years it increased from 48.6 per 100,000 to 99.4 per 100,000 during the same time period. The high mortality rates, which are clearly increasing in TT 46, and the observed disparities by ancestry reported herein suggest that genomic assessment of the mutational spectra as well as genetic ancestry analysis is warranted in TT. This is an ideal environment to examine associations between genetics, incidence, and disease aggressiveness since there are fewer disparate mitigating factors.

The observed differences in BC incidence and mortality by geography may be an indication that differential spatial access to timely and appropriate care might impact cancer diagnosis, prognosis, and mortality rates in this Caribbean setting. Although there is universal healthcare coverage in TT, patients are required to obtain referrals from a physician prior to accessing treatment 47. While there are no geographic boundaries in TT per se that limit access to the public medical facilities that provide cancer care and at minimum, one hospital and several clinics are located within each RHA catchment area, there are only four licensed oncologists (none in Tobago) serving the entire population 48. Furthermore, the estimated travel time in TT to an oncology center is between 0.5 and 9 h with <1 h for 26.6% of the population versus in the U.S. (e.g.,) where it is <1 h for 92% of the population 49. This might be a major contributing factor to the geographic disparities. Numerous studies report an inverse association between travel time and cancer care 50–55. However, in TT the highest incidence and mortality rates are in areas closest to cancer centers. Further examination of the association between spatial access, distance- and travel time to cancer care facilities, and BC outcomes in TT is needed.

This study has some limitations that should be considered in the interpretation of our findings. First, there were missing data, particularly related to patients’ ancestry, which highlights the need for more thorough data collection by cancer registry. Another consideration is that this study was based on retrospective analysis of an anonymous dataset; there was no way to supplement any missing data. Another limitation of this study was that we did not evaluate BC screening patterns. Therefore, the observed disparities in mortality may be due to disparities in screening and possibly treatment, which may be dependent upon a patient’s place of residence, differences in resource allocation by RHAs and the overall cancer care received. As a result of this, we cannot verify that cancer patients seek cancer care at medical centers within their region of residence, which is another limitation of this study. Patient demographic information was self-reported and may not reflect the entire picture. In particular, ancestry was self-reported, without the benefit of genomic screening for admixture markers. The absence of data on reproductive BC risk factors (e.g., age at first birth, and body mass index (BMI) was also a limitation. The association between BMI and BC mortality is well established and studies have shown significantly higher BMI among women of African ancestry, compared to other racial/ethnic groups 56, which could have contributed to the differences in BC mortality and survival by ancestry observed in this study.

Despite limitations, this work has important implications. This study is the first to examine associations among ancestry, geography, and BC incidence, mortality, and survival in Trinidad and Tobago. The importance of this work is underscored by the fact that the BC incidence and mortality rates are among the highest in the Caribbean 13–15,57 and the world even though 1–3 TT has the largest healthcare budget in the Caribbean. In low- and middle-income countries, 18% of BC cases are attributable to modifiable causes such as alcohol intake, physical inactivity, and postmenopausal overweight and obesity 58. Hence, prevention activities, which incorporate the adoption of a healthier lifestyle, including diet, could impact BC incidence in TT. Nevertheless, early detection to improve survival might have the greatest impact in BC control in TT and efforts to promote such endeavors are needed. This study also provides an ancestral and geographical context from which resource allocation decisions can be made. The findings presented here highlight the need for similar studies in the other Caribbean island nations. In addition, this study highlights the need for targeted outreach efforts by ancestry and geography so as to reduce the documented disparities. Given the size of Trinidad and Tobago, the disparities reported herein suggest a need for genetic/genomic studies to determine whether these are ancestry-based genomic alterations driving incidence, mortality, and treatment resistance. This study also highlights the need for research to understand why the highest rates of incidence and mortality are in close proximity to cancer centers and biomedical institutions, given that TT has an equal access to care model.

In conclusion, we present evidence that there are associations between ancestry, geography, and BC incidence, mortality, and survival in TT. Medical interventions that seek to improve oncology services nationally will fail to narrow these disparities since there might exist population-level social inequalities, and genomic differences, which can contribute to our findings. Our findings indicate a need for a national dialog on these disparities and the deployment of equal access and utilization of *quality* cancer care, early detection, genetics and genomics research, molecular BC subtyping, targeted outreach, and improved therapeutics to reduce BC disparities.
